# Huge typical pulmonary carcinoid presented with gigantism syndrome

**DOI:** 10.1007/s11748-020-01445-9

**Published:** 2020-07-28

**Authors:** Morris Beshay, Federico Gutierrez, Beatrice Ariane Windmöller, Christina Förster, Fritz Mertzlufft

**Affiliations:** 1 Department of General Thoracic Surgery, Division of General Thoracic Surgery, Protestant Hospital of Bethel Foundation, Burgsteig 13, 33617 Bielefeld, Germany; 2 Department of Cell Biology, University of Bielefeld, Bielefeld, Germany; 3 Institute of Pathology, KRH Hospital Nordstadt, Haltenhoffstrasse 41, Hannover, Germany; 4 Department of Anesthesia and Intensive Care, Protestant Hospital of Bethel Foundation, Bielefeld, Germany

**Keywords:** Typical pulmonary carcinoid, Lung cancer, Gigantism

## Abstract

A 27-year-old male patient presented with cough and right-sided, light thoracic pain. His physical appearance showed typical features of gigantism. Subsequently, further diagnostic work-up showed elevated level of growth hormone and a huge tumor of the right lung, identifying a typical pulmonary carcinoid tumor (TPCT). Curative surgery was performed leading to normalization of the elevated growth hormone levels few days after surgery. Two- and five-year follow-up showed no signs of recurrence. Respected to tumor size, we determined the largest TPCT to be reported in medical literature history.

## Introduction

Pulmonary carcinoids were initially labeled as adenomas of the bronchial system [1]. Although most classic carcinoids are located centrally, a few are found in the periphery of the lung [2]. Carcinoid tumors of the lung, such as of the intestinal tract, are derived from neuroendocrine Kulchitsky cells located within the bronchial or intestinal mucosa. These tumors may produce a number of peptides, such as serotonin, for example. However, unlike intestinal manifestations, association of lung carcinoids with a carcinoid syndrome is rare (2.6%). Many reports showed long-time survival after surgical resection of TPCT, one of them proclaims an excellent 10-year survival rate of 87% [3].

In our case, we report an exceptional huge TPCT in a young male patient with gigantism syndrome.

## Case

A 27-year-old, non-smoking, 197 cm tall, 118 kg weight male patient presented himself at an external medical practice with symptoms including cough and right-sided, thoracic pain. His past medical history was uneventful. Next to signs of gigantism, the physical examination determined weak breath sounds of the right lung. Furthermore, the patient suffered from augmentation of the facial configurations, large extremities with oversized hands and feet, with no correlation to the height of his parents. Even though laboratory investigations showed normal results, growth hormone levels were found to be massively elevated: 27.6 µg/l (normal 0.1–4.1 µg/l).

**Fig. 1:**
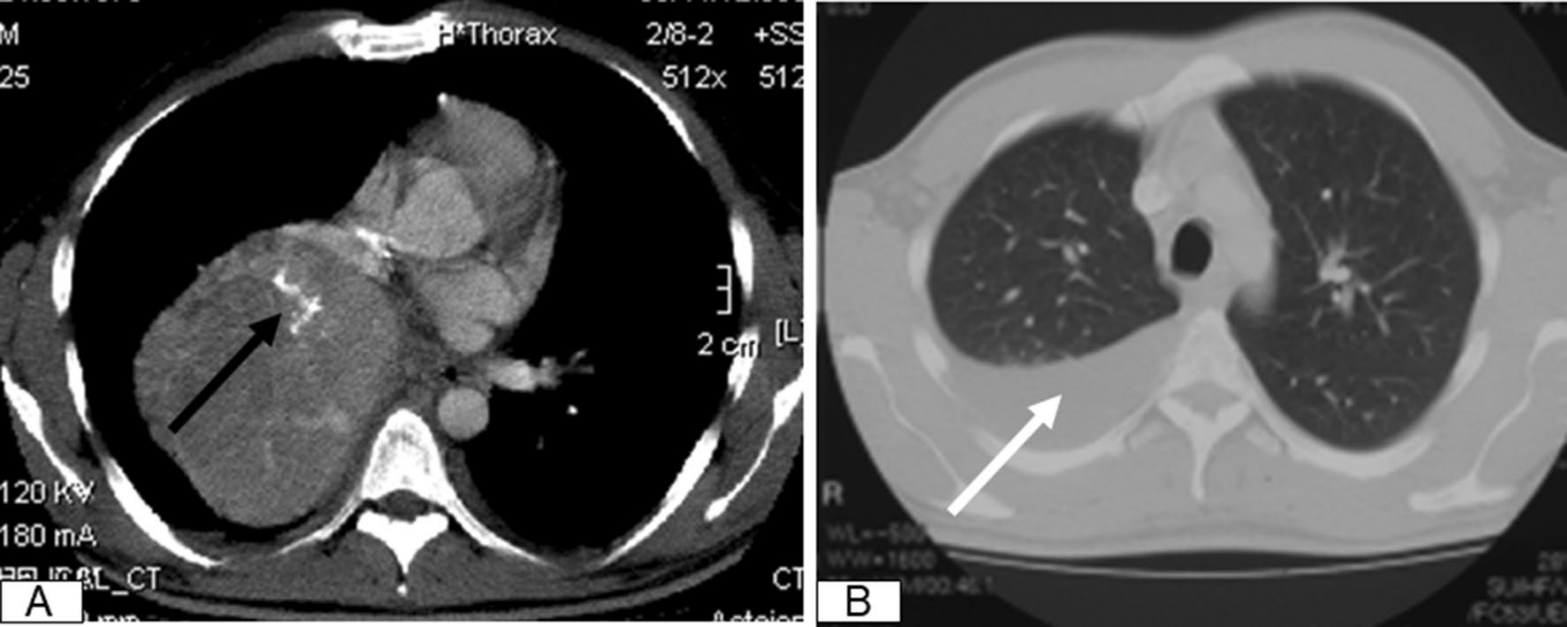
CT scan of the chest, showing the large tumor on the right side with calcification inside (black arrow) (**a**), accompanied pleural effusion (white arrow) (**b**)

**Fig. 2:**
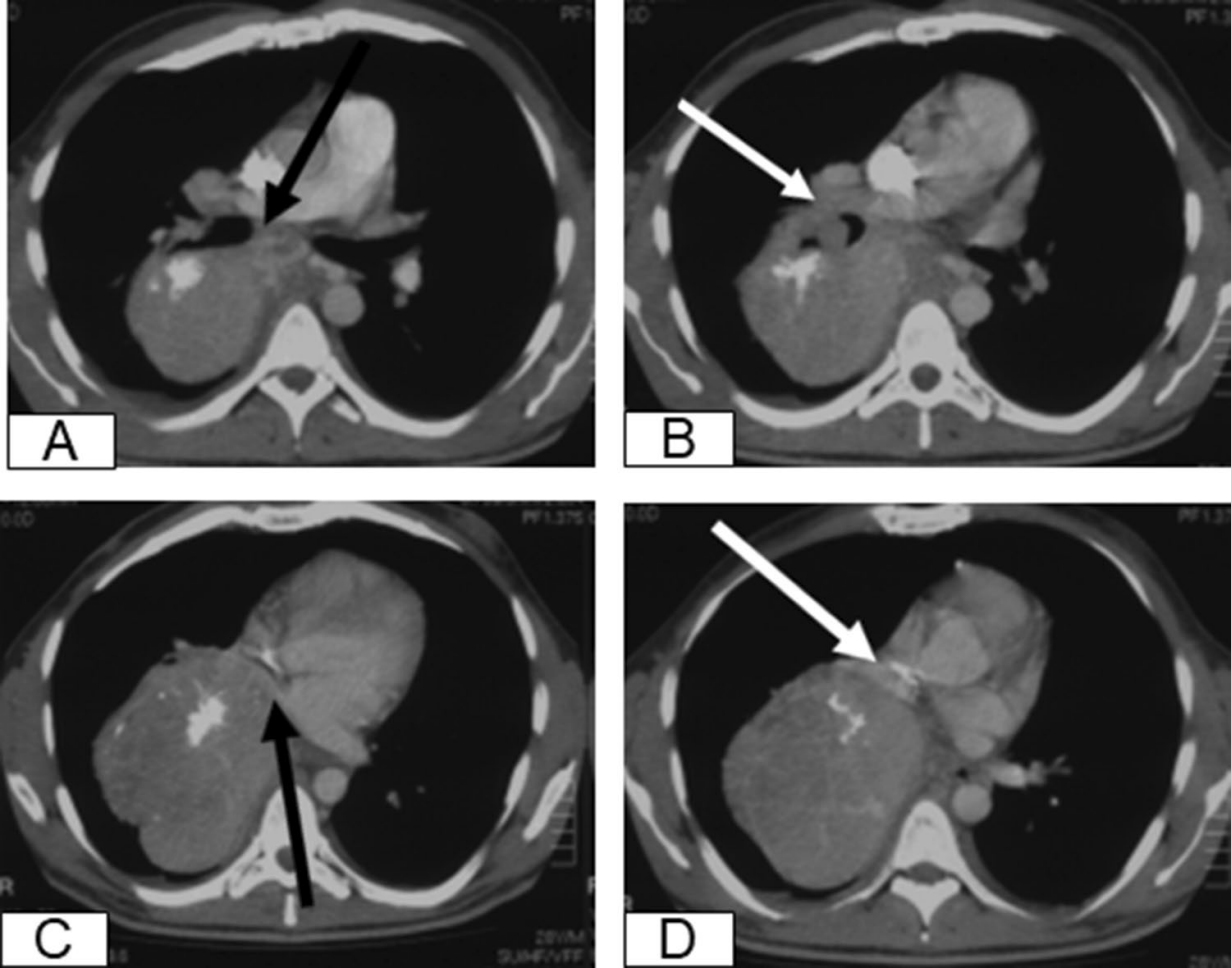
CT scan of the chest, showing close relationship to the right main bronchus (black arrow) (**a**), close relationship to the right main pulmonary artery (white arrow) (**b**), compressed upper lung vein (black arrow) (**c**), totally occluded right lower lung vein (white arrow) (**d**)

**Fig. 3:**
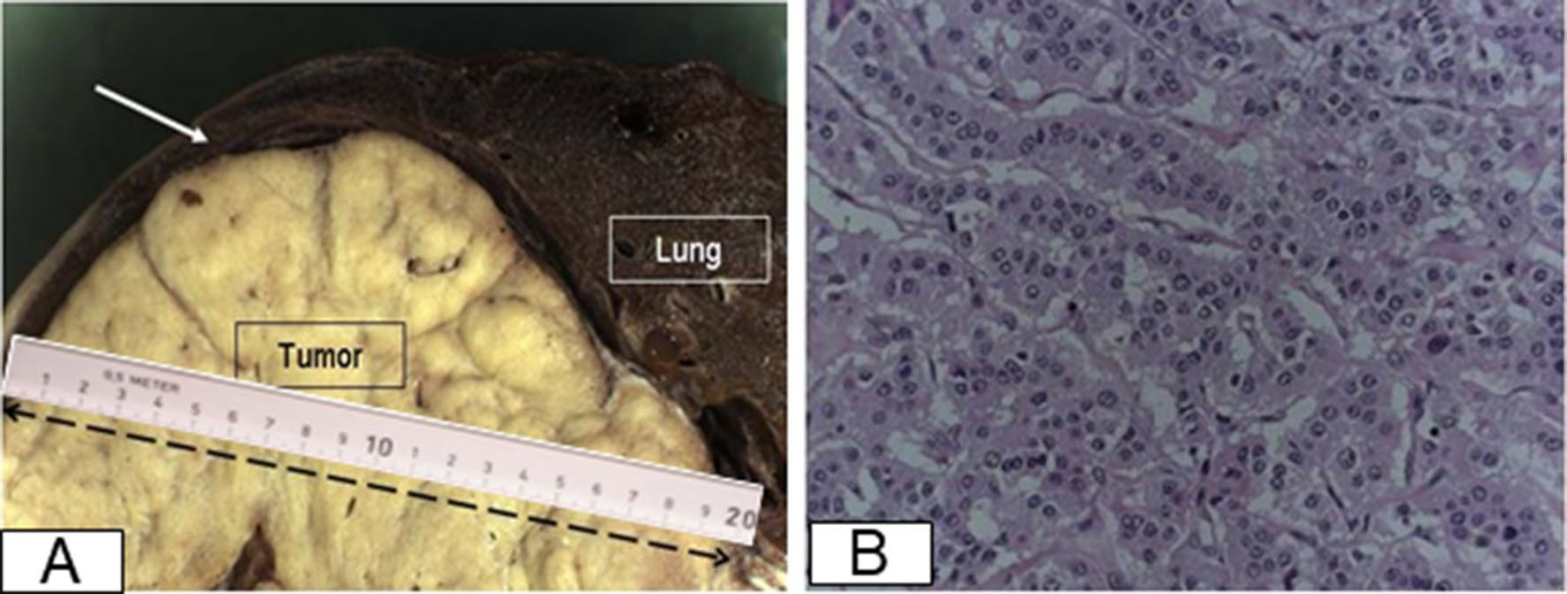
A macroscopic picture showing the tumor with 17 cm in diameter (**a**), a microscopic picture showing typical carcinoid tumor with rarely mitosis (0–1/2 mm^2^) (**b**)

A subsequent CT scan revealed a huge tumor in the right lower lobe accompanied with calcifications as well as moderate pleural effusion (Fig. 1). The tumor seems to invade all 3 lobes of the right lung, the posterior wall of the right main bronchus, both the upper and lower lung veins, as well as the right main pulmonary artery after the origin of the upper arterial trunk (Fig. 2). Body plethysmography showed FEV1 of 85% and VC of 90%. Bronchoscopic examination showed complete obstruction of the right intermediate bronchus; however, the endobronchial biopsy showed no diagnosis. Therefore, we decided to perform a surgical biopsy. The exploration showed a huge, solid tumor, mainly located within the lower lobe, but infiltrating the middle lobe as well as two more segments of the upper lobe. Further exploration showed adherence of the tumor on the posterior wall of the right main bronchus, obliteration of the right lower lung vein and near total obliteration of the middle and upper lung veins. A frozen section revealed a TPCT. In this situation, right pneumonectomy with systematic mediastinal lymph node dissection was performed though prolongation of the small incision to an antero-lateral thoracotomy. In fact, the tumor measured 20 × 13 cm in diameter (Fig. 3a). The histopathological work-up showed tumor invasion of all three lobes of the right lung and confirmed the invasion of the posterior wall of the right main bronchus, the lower, middle as well as the upper lung veins. There were thick adhesions not only in the fissure but also to the chest wall and the diaphragm. In the para vertebral region, local pleurectomy was done because of thick adhesions between the parietal pleura and the lower lobe. Systematic lymph node dissection was done with removal of the paratracheal lymph nodes, the infracarinal, the hilar as well as the paraoesophageal lymph nodes.

Histological specimen revealed typical carcinoid tumor with peripheral calcification indicating slow growing behavior (Fig. 3b). Fortunately, no hilar or mediastinal lymph node metastases were detected. Furthermore, the tissue was analyzed immunohistochemically revealing a positive reaction for AE1/AE3 and synaptophysin; however, low proliferation antigen (1% in the cell nucleus) Ki67 (MIB 1) was revealed. Further, mitosis activity was seen in 0–1/2 mm^2^ (Fig. 3). Further testing determined negative reaction for chromogranin A. All these findings are characteristics for diagnosis of TPCT. According to WHO classifications in 1997, pathological tumor staging indicates in this case pT2, pN0, M0 (stage IB); whereas the WHO’s classification 2017 indicates pT4, pN0, M0 (stage IIIA).

The postoperative follow-up was uneventful. Growth hormone returned to normal levels (3.0 µg/l) within few days after surgery. The patient was discharged on the 11th postoperative day in an excellent physical condition. To date, the patient is recurrence-free and under follow-up (2- and 5- and 10-year follow-up was done performing CT scans).

## Discussion

Typical pulmonary carcinoid syndrome is a rare occurrence. Patients with TPCT almost never exhibit symptoms. TPCT are known to be active endocrinal tumors, although it is a very rare phenomenon. Hormone production is seldom but gigantism, acromegaly or Cushing’s syndrome for example is the commonest in the literature. Other rare endocrinopathies have been reported in such patients [4]. In our case, the tumor produced high amounts of growth hormone since puberty, which was produced over many years because of an active pulmonary endocrinal tumor. Moreover, pre-operative cranial CT scan showed normal size of the pituitary gland. Due to the increased production of growth hormone, we postulate that the cause of gigantism in this patient was caused by this active TPCT. Postoperative testing confirmed our thesis by showing a rapid decrease of growth hormone levels. Gigantism syndrome and the presence of calcification in the tumor tissue confirm the slow rate of tumor growth. Even though the occurrence of huge carcinoid before or during puberty is rarely seen in young patients, it may indicate yet a bad prognosis. However, radical curative resection of the tumor has been described in the past [4, 5].

Carcinoid tumors do not present specific radiological signs. Huge-size carcinoid tumors could be mistaken as benign lesions, for example fibroma or lipoma. Nevertheless, patients with typical carcinoid tumors have an excellent prognosis, with up to 87% survival rate at 10 years and 74% at 15 years [3–5]. According to the new pathological classification of lung cancer, carcinoid tumors belong to the group of neuroendocrinal tumors, and represent one end of the spectrum, whereas small cell lung cancer (SCLC) represents the other end. Other tumor entities in this spectrum are atypical carcinoid as well as large cell neuroendocrine carcinoma (LCNEC). Although these entities share probably the same cell origin, the prognosis is completely different in favor (better prognosis) of typical carcinoid tumors [6]. Therefore, histological diagnosis is always mandatory. Nevertheless, it is mandatory to treat these tumor identities in a curative way through anatomical resection and systematic lymph node dissection. Every effort has to be taken, to avoid performing pneumonectomy due to its late complications, especially in young adults [7]. In our case, a pneumonectomy was needed due to tumor infiltration of all three lobes especially in the great fissure. Moreover, the tumor has invaded the posterior wall of the right main bronchus as well as the distal part of the right main pulmonary artery with sub-complete occlusion of the right upper lung vein and complete occlusion of the right lower lung vein. In this situation, there was no any other way to treat this patient in a curative way without performing a right pneumonectomy.

In summary, huge typical carcinoid tumors are extremely rare, proclaiming a surgical challenge. With respect to the literature, this is the first report about carcinoid tumors associated with gigantism syndrome. Furthermore, our report seems to be the first case with huge carcinoid tumors in a young male, who was successfully treated surgically in curative intend. In the literature one patient with carcinoid tumor associated with acromegaly has been reported [8]. Synchronous carcinoid tumors are not only rare but also have been reported. Genetic factors involving the development of typical carcinoid tumors remain unclear. New reports on the genetic identity of TCT are emerging with respect [9].

There exists no consensus on how to follow up patients after surgery for a typical carcinoid. We recommend a CT scan once every 5 years at the latest. We suggest that, in case of gigantism, chest X-ray should be added to the investigation methods to identify such lesions and to plan a useful therapy. Typical carcinoid tumors are potentially curable, even when they reach a large size. The management of such cases requires careful investigation, planning, and an aggressive treatment strategy.

## Conclusion

Typical carcinoid is a slowly growing tumor, which could reach huge sizes. It is usually physiologically inactive but hormonal activities are rare known. In this case, the tumor produced a significant amount of growth hormone, which affected the patient’s configuration information of a gigantism. The tumor was surgically removed successfully in a curative intention. Long-term follow-up was uneventful.
